# Single-Cell Transcriptomic Analysis of the Mouse Pancreas: Characteristic Features of Pancreatic Ductal Cells in Chronic Pancreatitis

**DOI:** 10.3390/genes13061015

**Published:** 2022-06-05

**Authors:** Xiaotong Mao, Shenghan Mao, Lei Wang, Hui Jiang, Shunjiang Deng, Yuanchen Wang, Jun Ye, Zhaoshen Li, Wenbin Zou, Zhuan Liao

**Affiliations:** 1 Department of Gastroenterology, Changhai Hospital, The Second Military Medical University, Shanghai 200433, China; xiaotong_mao@163.com (X.M.); maoshenghan@smmu.edu.cn (S.M.); onerain@126.com (L.W.); shunjiangdeng90@163.com (S.D.); wangyuanchen@smmu.edu.cn (Y.W.); yejunn88@163.com (J.Y.); zhaoshenli@smmu.edu.cn (Z.L.); 2 Shanghai Institute of Pancreatic Diseases, Shanghai 200433, China; 3 Department of Pathology, Changhai Hospital, The Second Military Medical University, Shanghai 200433, China; jianghui5131@163.com

**Keywords:** chronic pancreatitis, single-cell analysis, transcriptome, mouse model, caerulein, mesenchymal cells, pancreatic ductal cells

## Abstract

Chronic pancreatitis (CP) is a fibroinflammatory disorder of the pancreas. Our understanding of CP pathogenesis is partly limited by the incomplete characterization of pancreatic cell types. Here, we performed single-cell RNA sequencing on 3825 cells from the pancreas of one control mouse and mice with caerulein-induced CP. An analysis of the single-cell transcriptomes revealed 16 unique clusters and cell type-specific gene expression patterns in the mouse pancreas. Sub-clustering of the pancreatic mesenchymal cells from the control mouse revealed four clusters of cells with specific gene expression profiles (combinatorial expressions of *Smoc2*, *Cxcl14*, *Tnfaip6*, and *Fn1*). We observed that immune cells in the pancreas of the CP mice were abundant and diverse in cellular type. Compared to the control, 547 upregulated genes (including *Mmp7*, *Ttr*, *Rgs5*, *Adh1*, and *Cldn2*) and 257 downregulated genes were identified in ductal cells from the CP group. The elevated expression levels of MMP7 and TTR were further verified in the pancreatic ducts of CP patients. This study provides a preliminary description of the single-cell transcriptome profiles of mouse pancreata and accurately demonstrates the characteristics of pancreatic ductal cells in CP. The findings provide insight into novel disease-specific biomarkers and potential therapeutic targets of CP.

## 1. Introduction

The pancreas is a bifunctional organ that produces both digestive enzymes and endocrine hormones. It is comprised of two main cellular compartments: the exocrine pancreas, primarily consisting of acinar and ductal cells, and the pancreatic islets, composed of no less than five distinct hormone-secreting cell types, including α cells, β cells, γ cells, δ cells, and ε cells. Chronic pancreatitis (CP) is a progressive fibroinflammatory syndrome of the pancreas that causes irreversible tissue destruction, leading to a permanent loss of pancreatic functions [1]. The salient pathologic features of the disease include glandular atrophy, extensive fibrosis, and inflammatory infiltration, as well as ductal dilatation and pancreatic calcification [2]. The progression of CP is accompanied by alterations in the cellular composition and the unique biological functions of specific cell types.

Single-cell RNA sequencing technology (scRNA-seq) has provided new insights into the diversity of pancreatic cell types and the plasticity of the pancreas. Several studies have investigated single-cell transcriptional profiles of the human and mouse pancreas [3,4,5], type 2 diabetes human islets [6,7,8], and pancreatic cancer [9,10,11]. Based on these studies, the precise pancreatic cell types and the cell type-specific gene expression signatures of diabetes progression, as well as intratumor heterogeneity and the progression of pancreatic cancer, are now becoming understood. Recently, Tosti et al. utilized single nuclear RNA sequencing (snRNA-seq) to generate transcriptome profiles of the pancreas from healthy neonates, healthy adults, and CP patients [12]. Single-cell analyses by Blobner et al. demonstrated two unique clusters of acinar cells in human CP [13], and Lee et al.’s study revealed distinct immune microenvironments in human hereditary and idiopathic CP [14]. These findings contribute to our understanding of the complex pancreatic genomic landscape and the cellular foundations of CP. 

Nevertheless, due to challenges in the preparation of pancreatic single-cell suspensions, the available single-cell reference map of diverse pancreatic cell types, especially in CP samples, remains limited. A well-developed model of CP that recapitulates human disease is the repetitive administration of caerulein injections in mice [15]. Here, scRNA-seq technology was utilized to generate transcriptional profiles of pancreata from healthy and caerulein-induced CP mice. The data demonstrated the heterogeneity of the mouse pancreas at a high resolution and revealed the cellular composition and transcriptome changes in pancreatic cells in experimental CP.

## 2. Materials and Methods

### 2.1. Mice

C57BL/6 male mice (20–22 g, eight weeks old) were purchased from Lingchang Biotechnology Co., Ltd. (Shanghai, China). The mice were randomly assigned to two groups (four mice in each group). To induce CP, mice were injected with caerulein (50 μg/kg/h) intraperitoneally six times per day at one-hour intervals, three days per week, for six weeks. The control mice were injected with normal saline (NS) in lieu of caerulein. The mice were then sacrificed and examined six days after the final injection. All animal experiments were performed with the approval of the Animal Care Committee of Second Military Medical University.

### 2.2. Tissue Digestion

The digestion buffer was prepared in DMEM/F-12 (11039-021, Gibco, Grand Island, NY, USA) and contained 100 units/mL of collagenase type I (Worthington Biochemical, Lakewood, NJ, USA), 125 units/mL collagenase type IV (Thermo Fisher Scientific, Grand Island, NY, USA), and 25 units/mL DNase type I (Sigma-Aldrich, St. Louis, MO, USA). Pancreata from one control mouse and two CP mice were enzymatically digested into a single-cell suspension, respectively. Note that the cell number yield from CP mice was relatively low; two samples from CP mice were pooled to obtain enough cells for sequencing. Briefly, freshly dissected tissue was placed in a new 10-cm culture dish and cut into pieces with a clean razor blade. After washing twice with phosphate-buffered saline (PBS), each sample was moved to a 15-mL tube containing prewarmed 37 °C digestion buffer comprising 1% fetal bovine serum, respectively. The tubes were incubated on a shaker for 40 min at 37 °C. Cell debris was filtered out with a 30-μm mesh filter, and the cells were then resuspended and washed three times in PBS. Single cells were then resuspended in 100 μL of PBS in preparation for a subsequent single-cell library construction. A Cellometer Auto 2000 Cell Viability Counter (Nexcelom Bioscience, Lawrence, MA, USA) was used to assess the cell viability.

### 2.3. Single-Cell cDNA Library Preparation and Sequencing

We used the BD Rhapsody System (BD Biosciences, Piscataway, NJ, USA) to capture single cells derived from two pancreas samples. The resultant single-cell suspension was washed in PBS (free of calcium and magnesium; 400 μg/mL bovine serum albumin). The final concentration was 600–800 cells/μL. Following the manufacturer’s instructions, an appropriate volume of pancreatic cells was loaded on a BD Rhapsody cartridge and incubated at room temperature. Cell Capture Beads were prepared and loaded on the cartridge so that one bead would bind to one cell. The cartridge was washed, and the cell lysis buffer was added. The beads hybridized with poly-adenylated RNA molecules were then collected for cDNA library construction. scRNA-seq and bioinformatic data analysis were performed by Shanghai Novelbio Ltd (Shanghai, China).

### 2.4. Bioinformatics Analyses

The adaptor sequences were processed by fastp with default parameter filtering, and the low-quality reads were removed [16]. UMI-tools was utilized for a single-cell transcriptome analysis to determine the cell barcode whitelist set by the cell; cell barcodes were captured by the BD Rhapsody platform and observed by a BD Scanner. Reads were mapped to the mouse genome by STAR aligner. Cell barcode UMIs were extracted from the bam file, and the cell expression counts were calculated for further analysis [17]. The Seurat package (version: 2.3.4) was applied according to the raw cell counts [18]. The gene expression values were log-normalized. Quality control was performed to filter out cells with less than 200 or more than 5000 genes expressed, as well as cells with more than 20% mitochondrial reads (Supplementary Figure S1). Seurat regression was performed based on the library size. PCA analysis was applied based on all HVGs (high variable genes); 20 dimensions were calculated, and the top 10 dimensions were used for Graph-Clust and a t-distributed stochastic neighbor embedding (t-SNE) analysis. A graph-based and k-mean-based clustering approach were performed using the FindCluster function in Seurat to identify the clusters. Wilcox *t*-tests were used for the FindAllMarkers function to identify the marker genes based on the cluster results above (log FC > 0.25; pct1 > 0.1; *p*-value adj < 0.05). Clusters of endocrine cells (high expression of *Chga*) [19] and clusters of mesenchymal cells (high expression of *Col1a1* and *Col3a1*) [4,19] were extracted for further sub-clustering analysis using Seurat with the same parameters, respectively. Gene Ontology (GO) enrichment analysis was performed with Metascape (http://metascape.org (accessed on 22 November 2020)) [20].

### 2.5. Histology and Immunohistochemistry

Mouse pancreata were removed and fixed in 4% paraformaldehyde (PFA) for 48 h. Pancreatic tissues were then embedded in paraffin. Five-micrometer tissue sections were loaded on slides for staining. Hematoxylin and eosin staining (H&E) and Masson’s trichrome staining (ab150686, Abcam, Cambridge, UK) were performed for histopathological evaluation. Human CP specimens were obtained from CP patients who underwent biopsy, and control specimens were obtained from patients with pancreatic neuroendocrine tumors who underwent surgery; they were available as formalin-fixed, paraffin-embedded tissue blocks. Patients gave their consent to participate in this study, which was approved by the Changhai Hospital Ethical Committee. Immunohistochemical staining was applied to the paraffin sections with antibodies against CD45 (ab10558, Abcam), F4/80 (#70076, Cell Signaling Technology, Danvers, MA, USA), CD3 (ab16669, Abcam), CD20 (MA5-13141, Invitrogen, Carlsbad, CA, USA), Cxcl14 (MAB866, Novus, Littleton, CO, USA), FN1 (15613-1-AP, Proteintech, Wuhan, China), MMP7 (10374-2-AP, Proteintech), and TTR (11891-1-AP, Proteintech).

## 3. Results

### 3.1. Induction of CP in Mice

**Figure 1:**
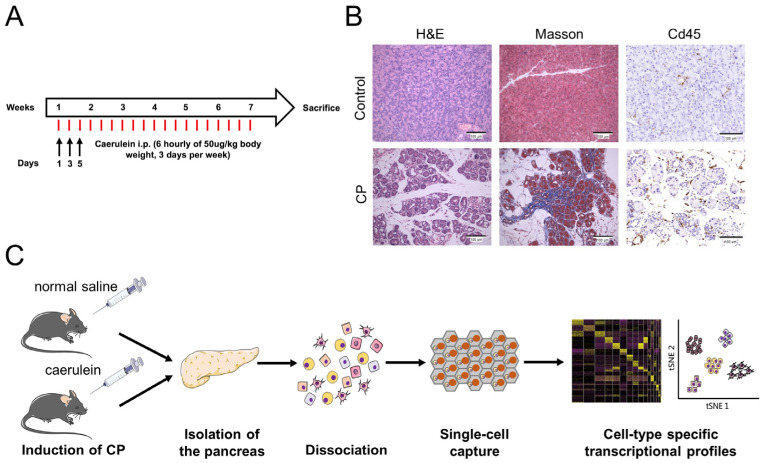
Induction of CP in mice. (**A**) Flow diagram of caerulein administration: six daily injections of caerulein given hourly on days one, three, and five of each week for six weeks. (**B**) H&E and Masson’s trichrome staining of pancreatic sections isolated from control and CP mice. The immunohistochemical analysis showed an extensive infiltration of Cd45 leukocytes. Scale bar = 100 μm. (**C**) Workflow for obtaining the single-cell transcriptome data from mouse pancreata.

CP was induced in eight-week-old C57BL/6 mice by an intraperitoneal injection of caerulein for six weeks (Figure 1A). Compared with control mice, CP mice exhibited a lower bodyweight gain and smaller-sized pancreas due to acinar cell loss. Histological analysis of the pancreata from caerulein-treated mice revealed pathological features of CP, including acinar destruction, inflammatory infiltration, duct dilatation, and fibrosis (Figure 1B). The freshly isolated pancreata from the control and CP mice were enzymatically dissociated into single-cell suspensions. Then, cDNA library generation was performed using the BD Rhapsody platform (Figure 1C).

### 3.2. Single-Cell Transcriptomes Recapitulate Pancreatic Cell Types

**Figure 2:**
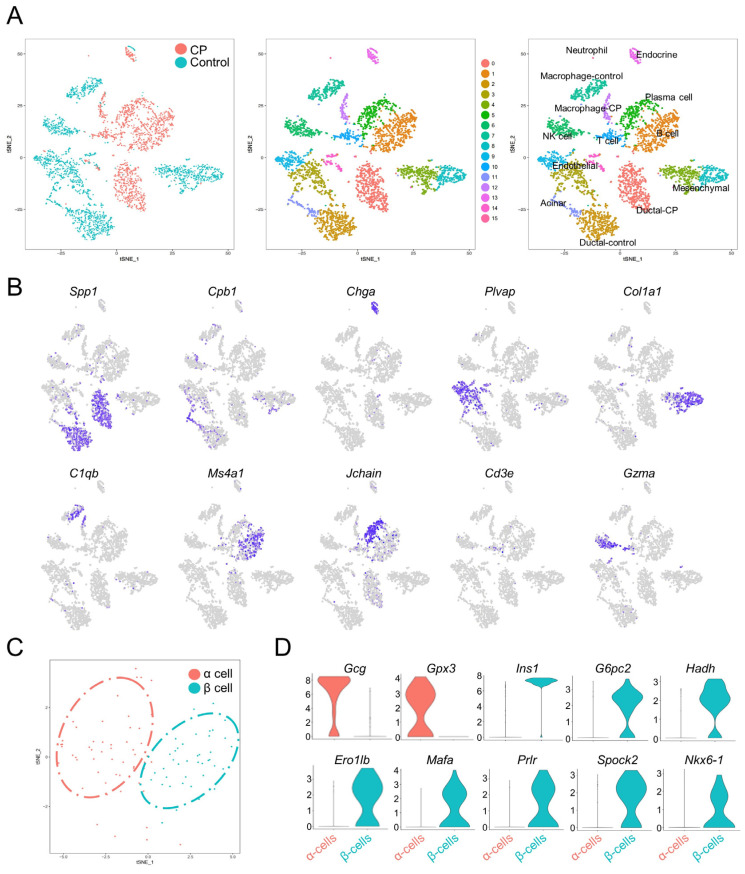
Single-cell transcriptomes recapitulate pancreatic cell types. (**A**) t-SNE plots of 3825 cells from mouse pancreata annotated by distinct tissue samples (left panel), and specific cell types distinguished by gene expression (right panel). (**B**) Heatmap illustrating specific markers in different pancreatic cell types. (**C**) t-SNE representation of α cells and β cells generated from sub-clustering the endocrine cell population, colored according to cluster assignments. (**D**) Violin plots illustrating the mRNA levels of representative marker genes in α and β cells. The y-axis displays the normalized read count.

The transcriptomes of 3825 single pancreatic cells were analyzed (1645 cells from CP mice and 2180 cells from the controls). t-SNE was utilized to project all cells onto two dimensions. This revealed 16 transcriptionally unique clusters (Figure 2A and Figure S2 and Supplementary File S1), including acinar cells (high expression of *Cpb1*, *Ctrb1*, and *Cpa1*); ductal cells (*Spp1* and *Krt18*) [3,19]; endocrine cells (*Chga*) [19]; mesenchymal cells (*Col1a1* and *Col3a1*) [4,19]; endothelial cells (*Plvap* and *Pecam1*) [7,19]; and immune cells (Figure 2B).

To determine the various endocrine cell types, a clustering analysis was performed on the endocrine cells, and the cells were separated into two clusters with distinct hormone expressions: α cells (*Gcg*) and β cells (*Ins*) (Figure 2C). Notably, α cells also showed high levels of *Gpx3*, while β cells expressed *G6pc2*, *Hadh*, *Ero1lb*, *Mafa*, *Prlr*, *Spock2*, and *Nkx6-1* (Figure 2D), which is consistent with a public database (http://bis.zju.edu.cn/MCA/gallery.html?tissue=Pancreatic-Islet (accessed on 18 May 2022)) and previous studies [3,4,6,7,8].

### 3.3. Subpopulation Identification of Pancreatic Mesenchymal Cells in Normal Mice

**Figure 3:**
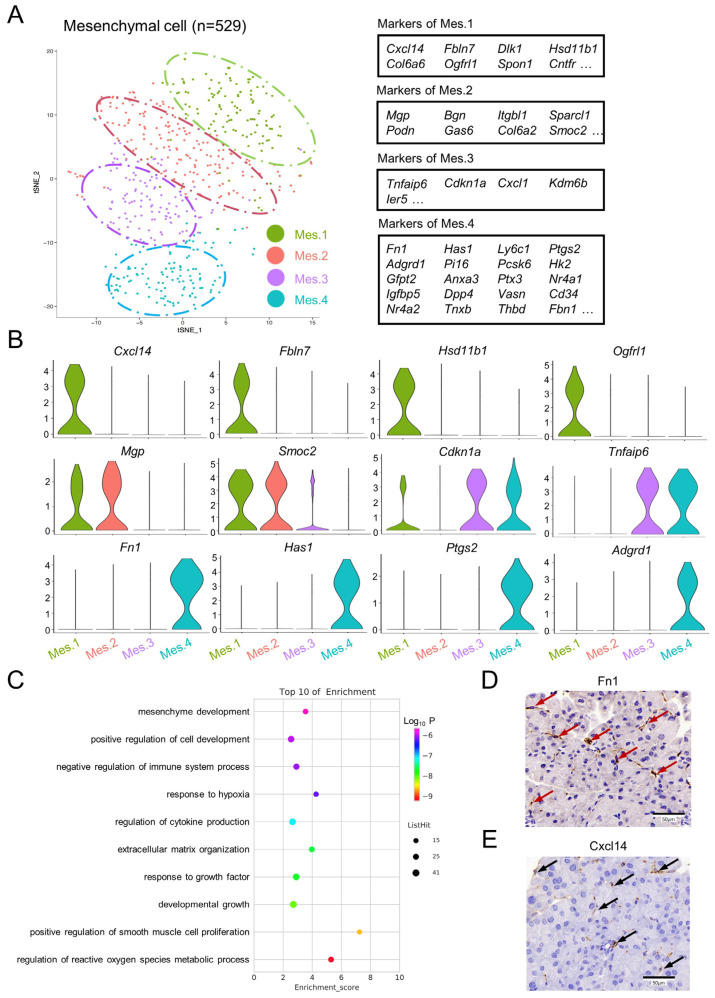
Subpopulation identification of pancreatic mesenchymal cells in normal mice. (**A**) t-SNE illustration of four subgroups identified from sub-clustering of the mesenchymal cell population, colored according to the cluster assignments (left panel). Tables demonstrating the top differentially expressed genes when comparing one subcluster to all other mesenchymal cells (right panel). (**B**) Violin plots showing representative marker genes in the subgroups. (**C**) Representative GO terms enriched for marker genes in Mes.4 based on a functional enrichment analysis. (**D**,**E**) Representative immunohistochemical images of pancreatic tissues from the control mice stained for Fn1 (red arrow) and Cxcl14 (black arrow). Scale bar = 50 μm.

In order to explore the heterogeneity within pancreatic mesenchymal cells, the clustering of mesenchymal cells was investigated in the control sample, and four discrete clusters were identified (Figure 3A and Supplementary File S2). Mesenchymal cell population 1 (Mes.1) and mesenchymal cell population 2 (Mes.2) were characterized by the expression of *Smoc2* and *Mgp*. In addition, Mes.1 specifically expressed *Cxcl14*, *Fbln7*, *Ogfrl1*, and *Hsd11b1* (Figure 3B). Mesenchymal cell population 3 (Mes.3) and mesenchymal cell population 4 (Mes.4) exhibited high levels of *Tnfaip6*. Mes.4 also had high expressions of *Fn1*, *Has1*, *Fbn1*, *Adgrd1*, *Ptgs2*, *Cd34*, *Pi16*, and *Ptx3* (Figure 3A,B). A GO analysis suggested that Mes.4 is involved in the positive regulation of smooth muscle cell proliferation, the negative regulation of the immune system process, and the regulation of the reactive oxygen species metabolic process (Figure 3C). The existence of Cxcl14-positive or Fn1-positive mesenchymal cells was further demonstrated by immunohistochemistry (Figure 3D,E).

### 3.4. Infiltration and Activation of Immune Cells in CP Mice

**Figure 4:**
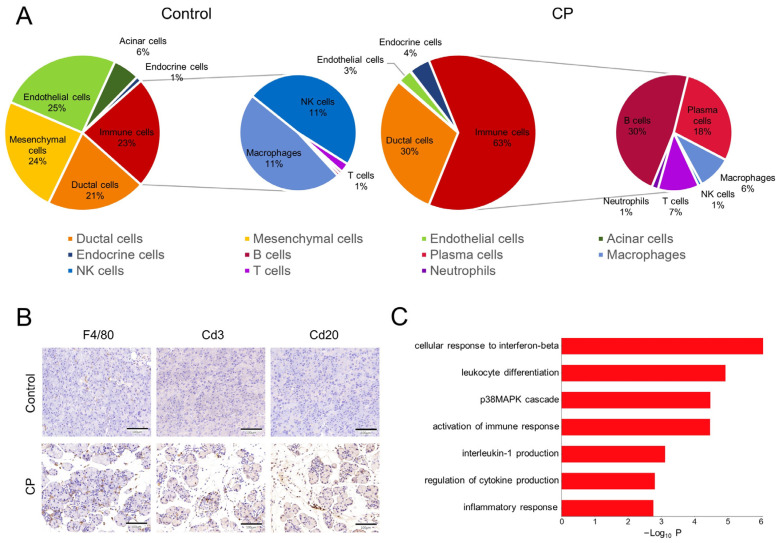
Alterations to immune cells in CP mice. (**A**) Pie charts showing the percentage of classified different cell types. The CP group had a higher proportion of immune cells and distinct immune cell profiles compared to the control group. (**B**) The immunohistochemical analysis showed the infiltration of F4/80 macrophages, Cd3 T cells, and Cd20 B cells in pancreatic sections from CP mice. Scale bar = 100 μm. (**C**) Significant GO terms enriched for upregulated genes of macrophages from the CP group.

Inflammatory infiltration is one of the main characteristics of CP. Our data showed a significantly increased proportion of immune cells in the CP mouse pancreas compared with the control pancreas (Figure 4A). An immunohistochemical analysis showed a strong infiltration of immune cells in the pancreatic sections from CP mice. F4/80-positive macrophages were found in the pancreatic sections from both the CP and control mice. Cd20-positive B cells and Cd3-positive T cells were observed in the CP pancreas but rarely detected in the control pancreas (Figure 4B). Notably, a higher *Il1β* expression level was observed in macrophages from the CP group than in macrophages from the controls. The GO analysis suggested that the upregulated genes of macrophages from the CP mice are involved in the cellular response to interferon-β, leukocyte differentiation, and the p38MAPK cascade (Figure 4C).

### 3.5. Ductal Cell Gene Expression Alterations in CP Mice

**Figure 5:**
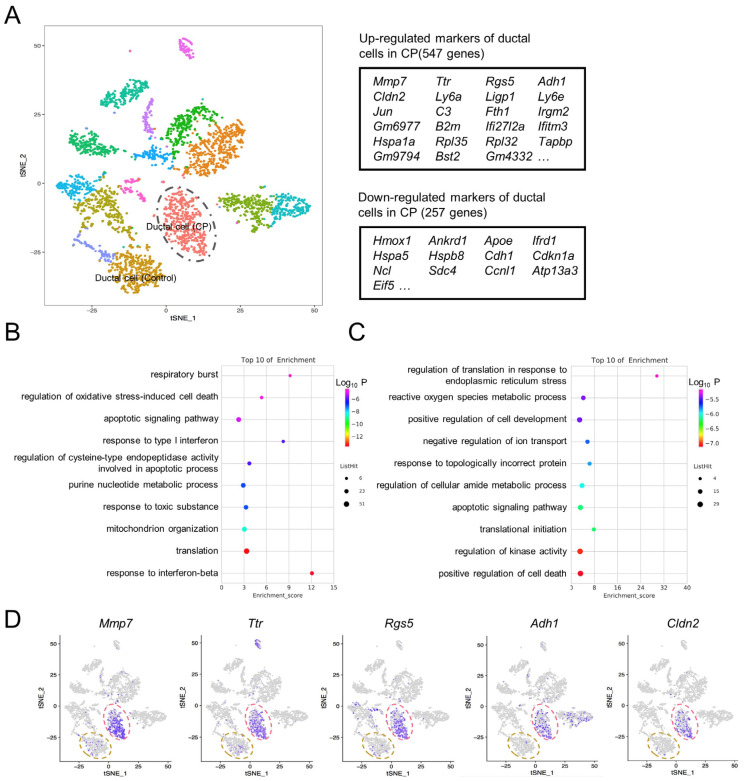
Gene expression alterations in ductal cells in CP mice. (**A**) The t-SNE map demonstrated that ductal cells from the CP group were completely separated from ductal cells from the control group (left panel). Tables denoting the top differentially expressed genes when comparing ductal cells in CP to ductal cells in the control (right panel). (**B**) GO terms enriched for upregulated genes of the ductal cells in CP. (**C**) GO terms enriched for downregulated genes of the ductal cells in CP. (**D**) t-SNE maps indicating marker genes of the ductal cells in CP. The dashed yellow circle represents ductal cells in the control group; the dashed red circle represents ductal cells in the CP group.

Pathological changes to pancreatic acinar cells in CP, including acinar cell degeneration and acinar-to-ductal metaplasia (ADM), have been well-studied [21,22]. In order to explore the gene expression features of ductal cells with regards to CP, the transcriptome differences between the two groups were compared, and a total of 547 upregulated genes and 257 downregulated genes were identified in the ductal cells of the CP group (Figure 5A and Supplementary File S3). The GO analysis demonstrated that 547 upregulated genes are involved in the response to interferon-β and the translation and regulation of oxidative stress-induced cell death (Figure 5B), while the 257 downregulated genes are involved in the positive regulation of cell death, response to topologically incorrect proteins, positive regulation of cell development, and the reactive oxygen species metabolic process (Figure 5C). Ductal cells from the CP pancreas exhibited markedly higher expression levels of matrix metallopeptidase 7 (*Mmp7*), transthyretin (*Ttr*), regulator of G-protein signaling 5 (*Rgs5*), alcohol dehydrogenase 1 (*Adh1*), and claudin 2 (*Cldn2*) (Figure 5D).

### 3.6. Immunohistochemical Analysis of MMP7 and TTR in Mouse and Human CP Samples

**Figure 6:**
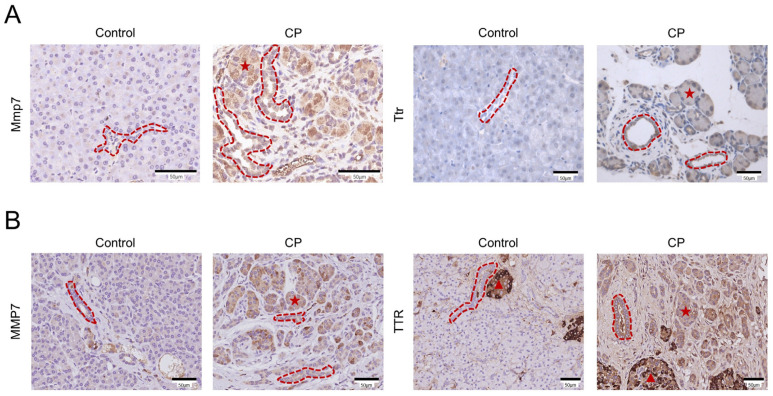
Immunohistochemical analysis of the MMP7 and TTR expression changes in mouse and human CP tissues. (**A**) Immunohistochemical staining of Mmp7 and Ttr in pancreatic tissues from the control and CP mice. (**B**) Representative immunohistochemical images of MMP7 and TTR in morphologically well-preserved pancreatic tissue (from a patient with a pancreatic neuroendocrine tumor) and pathological pancreatic tissue sections obtained from a patient with CP. The red dotted lines represent pancreatic ducts, the red pentagrams represent ADM structures, and the red triangles represent human islets of Langerhans. Scale bar = 50 μm.

To confirm the expression features of Mmp7 and Ttr in the ductal cells from CP mice, immunohistochemical staining of mouse pancreatic tissue sections was performed. As expected, immunohistochemistry showed increased levels of Mmp7 and Ttr in the pancreatic ducts of CP mice (Figure 6A). However, immunostaining revealed elevated expressions of Mmp7 and Ttr not only in the ductal cells but also in the ADM structures (Figure 6A). The expression patterns of MMP7 and TTR were also evaluated in vivo by performing an immunohistochemistry analysis of human CP samples and adjacent sections of morphologically normal pancreatic tissue obtained from patients with pancreatic neuroendocrine tumors (Figure 6B). Similarly, immunohistochemistry showed increased MMP7 and TTR expression in both the pancreatic ducts and the ADM structures of CP patients.

## 4. Discussion

As an essential component of both the digestive and endocrine systems, the human pancreas has a specific and complex cellular composition that changes (often markedly) during the course of a disease. CP is a fibroinflammatory syndrome of the pancreas characterized by recurrent episodes of pancreatic inflammation with variable severity and length. A comprehensive and accurate depiction of a normal pancreas and CP pancreas at the single-cell level can contribute to an improved understanding of the disease characteristics and diagnosis. However, human pancreas samples, which directly reflect the in vivo conditions of patients, are often difficult to obtain. One of the most frequently used methods to induce experimental CP is to perform a self-limited acinar cell injury by the repetitive injection of caerulein in mice. Although the clinical relevance of the caerulein-induced CP model remains unclear, the final common pathways of disease development appear similar [15]. In this study, scRNA-seq was utilized to explore the transcriptome profiles of the pancreata from healthy and experimental CP mice.

Using specific markers, most of the well-known pancreatic cell types were identified in the control group. Some uncommon endocrine cell types, such as γ, δ, and ε cells, were not separated from the whole endocrine cluster, perhaps due to the limited cell numbers. Notably, distinct subclusters of pancreatic mesenchymal cells were identified in the control group. Cells belonging to Mes.1 were distinguished by the upregulated expression of *Mgp*, *Smoc2*, *Cxcl14*, *Fbln7*, *Ogfrl1*, and *Hsd11b1*. *Hsd11b1* has been reported to be associated with glucocorticoid-induced insulin resistance [23] and the mediation of insulin release in pancreatic islets [24]. Notably, although *Hsd11b1* showed low expression levels in the islets, much higher levels were detected in intact micro-organs than purified α, β, or δ cells [25,26]. The current findings suggest that Mes.1 is the main source of Hsd11b1 in the pancreas. Cells belonging to Mes.4 expressed high levels of *Fn1*, *Tnfaip6*, *Has1*, *Fbn1*, *Adgrd1*, *Ptgs2*, *Pi16*, *PTX3*, and *Cd34*. The expression of Fn1, Ptgs2, and Cd34 has been reported in pancreatic stellate cells [27,28]. Notably, TNFAIP6 secreted from human adipose tissue-originated mesenchymal stem cells is able to ameliorate severe acute pancreatitis in mice by downregulating endoplasmic reticulum stress [29].

The immune cell profile of the CP group was quite different from that of the control. Macrophages and NK cells, cell types of the innate immune system, were detected in the control group, while B cells, T cells, and neutrophils were almost undetectable. In contrast, an enormous immune cell population was detected in the CP group, including B cells, T cells, plasma cells, macrophages, and neutrophils. According to Lee et al.’s study, T cells are the major immune cell type in the pancreas of CP patients [14]. The overabundant B cells and plasma cells in the CP group could be caused by species differences or modeling methods. Compared to macrophages from the control pancreas, macrophages from the CP pancreas exhibited a higher expression of *Il1β*, a proinflammatory factor associated with pancreatitis [30], suggesting that macrophages in the CP group exhibited M1-directed polarization. Notably, since we did not remove the circulating immune cells in the process of the preparation of the single-cell suspension, immune cells captured in the CP group may not fully correspond to the inflammatory cell profile of pancreatic tissues.

When comparing the transcriptomes of ductal cells between the two groups, ductal cells from the CP mice exhibited 547 genes with increased expression and 257 genes with decreased expression, which could be induced by long-term inflammation related to CP and immune responses. Notably, some acinar-specific marker genes, such as *Prss2*, *Try5*, and *Cpa1*, were discovered amid the downregulated genes of the CP ductal cells, possibly due to the ambient contamination from acinar cell lysis in the control group. As the most significantly downregulated gene, *Hmox1* encodes the rate-limiting enzyme for heme degradation—namely, heme oxygenase 1—which also serves as a key regulator of the inflammatory responses [31]. In animal models, elevated *Hmox1* expression ameliorates the course of pancreatitis [32]. Thus, a reduced expression of *Hmox1* in ductal cells of CP mice suggests a potential negative impact on the course of the disease.

The most significantly upregulated genes in the ductal cells of the CP group included *Mmp7*, *Ttr*, *Rgs5*, *Adh1*, and *Cldn2*. MMP7 is overexpressed in pancreatic intraepithelial neoplasia (PanINs) and pancreatic ductal adenocarcinoma (PDA) and has been found to promote the initiation and progression of PDA [33,34,35]; it is also correlated with the reduced survival and distant metastasis of PDA [35,36]. Under the physiological status, TTR is produced in the islets of Langerhans [37]. Our previous study showed that TTR is overabundant in pancreatic juice from patients with pancreatic cancer and CP [38]. In addition, TTR can promote the pancreatic insulin stimulus–secretion coupling of pancreatic β cells [39] and regulate the expression of glucagon in α cells [40]. The current study revealed, for the first time, that pancreatic ductal cells are another important derivation of TTR in CP. Interestingly, the immunohistochemical analysis showed an elevated expression of MMP7 and TTR in both the pancreatic ductal and ADM structures from both mice and patients with CP. As secreted proteins that are upregulated in the CP pancreas, MMP7 and TTR could be potential molecular markers for the diagnosis, intervention, treatment, and prognosis of CP.

RGS (regulator of G-protein signaling) proteins negatively regulate G-protein signaling, acting as GTPase-activating proteins for heterotrimeric G-protein α subunits, and RGS5 is a member of the G-protein signaling regulator family. Under normal conditions, the mRNA expression level of *RGS5* is very low in the pancreas [41]. *ADH1* encodes the α subunits of alcohol dehydrogenase 1 (class Ι), which catalyzes the NAD-dependent oxidation of alcohols [42]. Serving as tight junction proteins forming paracellular water channels of leaky epithelia, CLDN2 possesses low expression levels in tight junctions between normal pancreatic ducts cells. Notably, a nuclear factor (NF)-κB-binding site is in the *CLDN2* promoter [43], and some conditions associated with stress or injury elevate CLDN2 expression in some cells. Consistent with our findings, Cldn2 expression was found to be markedly upregulated in both acinar and ductal cells from CP cases in the study by Whitcomb et al. [44].

Compared with Hendley et al.’s study [45], we did not identify the subpopulations of the pancreatic ductal cells; this is considered to be due to the insufficient cell numbers. More recently, Ma et al. identified cell populations arising from ADM under conditions of chronic injury by combining lineage tracing and scRNA-seq technology [22]. Their data revealed that *Mmp7* and *Ttr* were also expressed at high levels in some intermediate cell clusters of ADM, which is consistent with our immunohistochemical findings. Acinar cells acquire ductal characteristics through the ADM process, and we speculate that ADM-derived cells could be included in the observed cluster of ductal cells in the CP group. Since the exocrine pancreas contains high levels of hydrolytic enzyme activities, pancreatic acinar cells more easily undergo autolysis than other cell types during the in vitro culture and dissociation steps. Compared with the 10× Chromium System (10× Genomics, San Francisco, CA, USA), the BD Rhapsody System utilizes microwells technology for single-cell capture (more than 200,000 microwells) and has a higher capture efficiency. Nevertheless, our data showed a small number of captured pancreatic acinar cells and a moderate sequencing quality.

In conclusion, this study presented a comprehensive investigation of the single-cell transcriptional profiles of mouse pancreata and revealed the heterogeneity of pancreatic mesenchymal cells. The data from mice with experimental CP accurately demonstrated the immune cell composition of the CP pancreas and the characteristic features of pancreatic ductal cells in CP. These findings provide insights into novel disease-specific biomarkers and potential therapeutic targets of CP.

## Data Availability

The sequencing data have been deposited in GEO under accession code GSE204844.

## Supplementary Materials

The following supporting information can be downloaded at https://www.mdpi.com/article/10.3390/genes13061015/s1: Figure S1: nUMI, nGene, and percentage of mitochondria genes in the transcriptome (percent.mito) of all cells from two samples. File S1: List of pancreatic cell-type markers and variable genes. Figure S2: Heat map of the top differentially expressed genes. File S2: List of differentially expressed genes in the subclusters of pancreatic mesenchymal cells. File S3: List of the up- and downregulated genes of pancreatic ductal cells from CP mice.
