# Supplementary figures and images for: Single-Cell Transcriptomic Analysis of the Mouse Pancreas: Characteristic Features of Pancreatic Ductal Cells in Chronic Pancreatitis
Source: Genes (Basel). 2022 Jun 5;13(6):1015. doi: 10.3390/genes13061015 (PMC9222509; doi:10.3390/genes13061015)

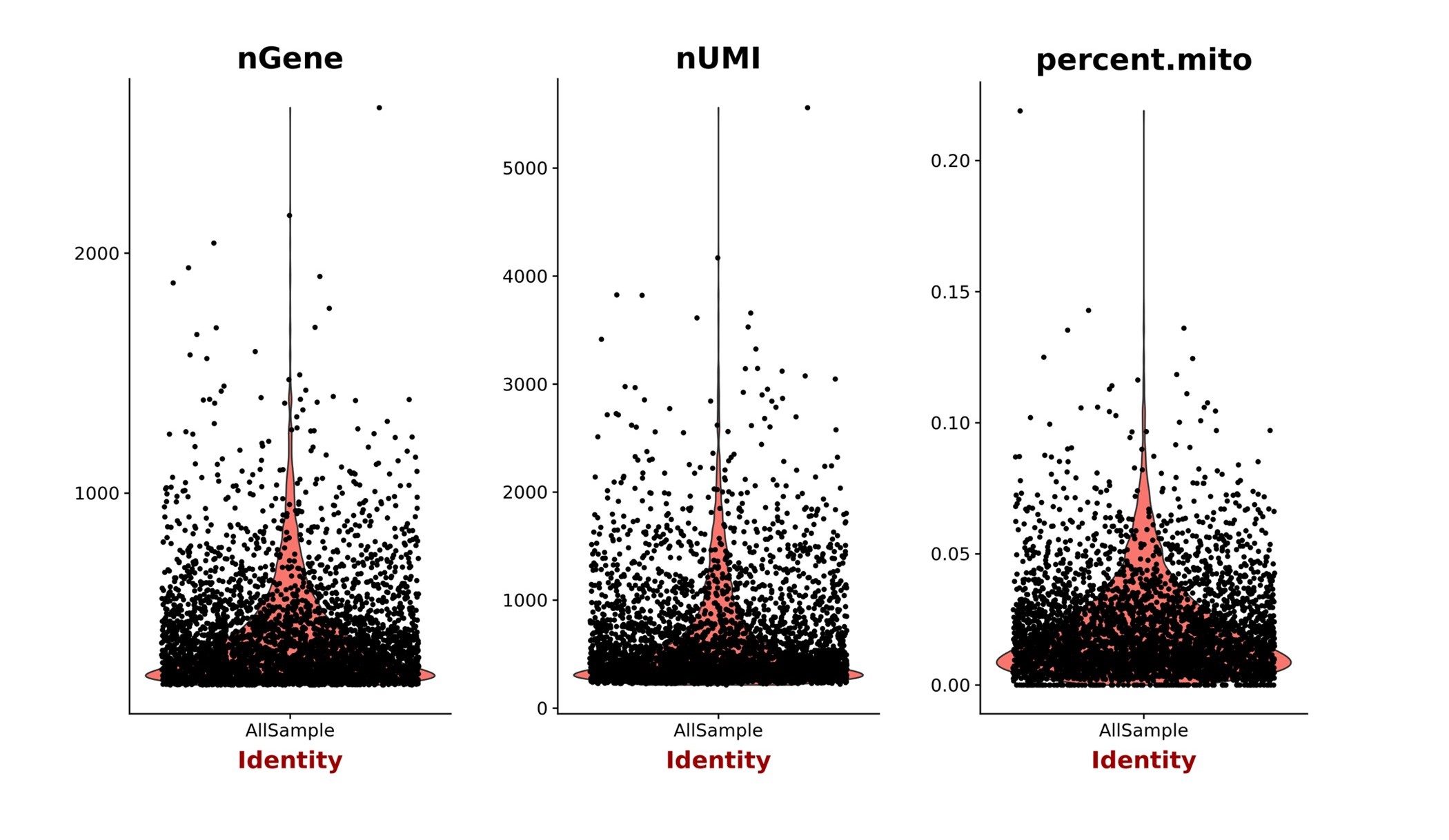

Supplement: Supplementary file 1 [file genes-13-01015-s001.zip › Supplementary_Figure 1.JPG]

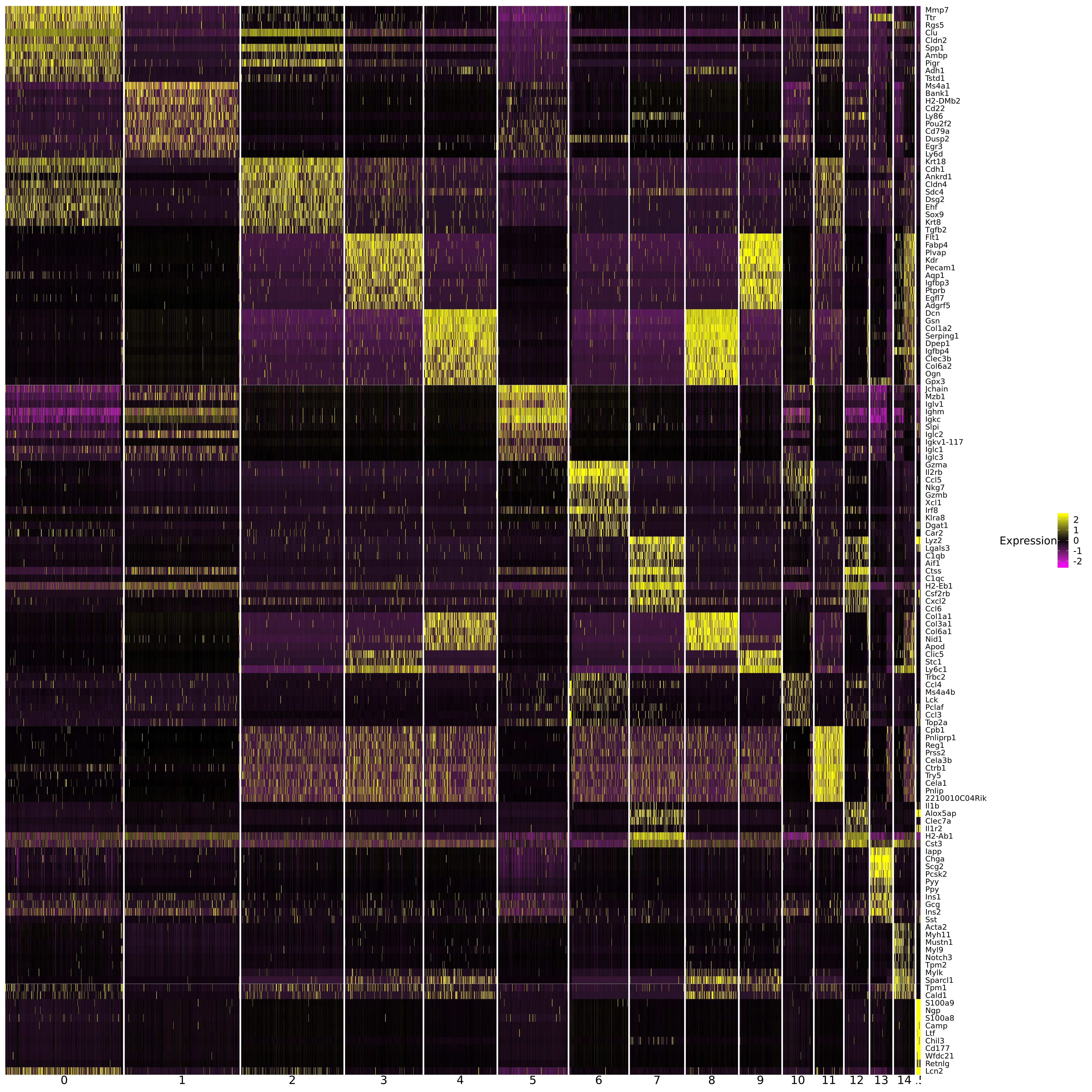

Supplement: Supplementary file 1 [file genes-13-01015-s001.zip › Supplementary_Figure 2.jpg]
